# High production of ectoine from aspartate and glycerol by use of whole-cell biocatalysis in recombinant *Escherichia coli*

**DOI:** 10.1186/s12934-015-0238-0

**Published:** 2015-04-15

**Authors:** Yong-Zhi He, Jiao Gong, Hai-Ying Yu, Yong Tao, Shan Zhang, Zhi-Yang Dong

**Affiliations:** State Key Laboratory of Microbial Resources, Institute of Microbiology, Chinese Academy of Sciences, Beijing, 100101 China; University of Chinese Academy of Sciences, Beijing, 100049 China; Department of Industrial Microbiology and Biotechnology, Institute of Microbiology, Chinese Academy of Sciences, Beijing, 100101 China

**Keywords:** Ectoine, *Halomonas elongata*, *Escherichia coli*, Whole-cell biocatalysis, Aspartate, Glycerol

## Abstract

**Background:**

Recently, the compatible solute 1, 4, 5, 6-tetrahydro-2-methyl-4-pyrimidinecarboxylic acid (ectoine) has attracted considerable interest due to its great potential as a protecting agent. To overcome the drawbacks of high salinity in the traditional bioprocess of ectoine using halophilic bacteria, various attempts have been made to engineer ectoine biosynthesis in nonhalophilic bacteria. Unfortunately, the yields of ectoine in these producers are still low and hardly meet the demands of large scale production. In this paper, the whole-cell biocatalytic process using aspartate and glycerol as substrates was tried for high production of ectoine in nonhalophilic bacteria.

**Results:**

The ectoine genes *ectABC* from the halophilic bacterium *Halomonas elongata* were successfully introduced into *Escherichia coli* K-12 strain BW25113 under the arabinose-inducible promoter. To our delight, a large amount of ectoine was synthesized and excreted into the medium during the course of whole-cell biocatalysis, when using aspartate and glycerol as the direct substrates. At the low cell density of 5 OD/mL in flask, under the optimal conditions (100 mM sodium phosphate buffer (pH 7.0), 100 mM sodium aspartate, 100 mM KCl and 100 mM glycerol), the concentration of extracellular ectoine was increased to 2.67 mg/mL. At the high cell density of 20 OD/mL in fermentor, a maximum titre of 25.1 g/L ectoine was achieved in 24 h. Meanwhile, the biomass productivity of ectoine is as high as 4048 mg per gram dry cell weight (g DCW)^−1^, which is the highest value ever reported. Furthermore, it was demonstrated that the same batch of cells could be used for at least three rounds. Finally, a total yield of 63.4 g ectoine was obtained using one litre cells.

**Conclusion:**

Using aspartate and glycerol as the direct substrates, high production of ectoine was achieved by the whole-cell biocatalysis in recombinant *E. coli*. Multiple rounds of whole-cell biocatalysis were established to further improve the production of ectoine. Our study herein provided a feasible biosynthesis process of ectoine with potential applications in large-scale industrial production.

## Background

**Figure 1:**
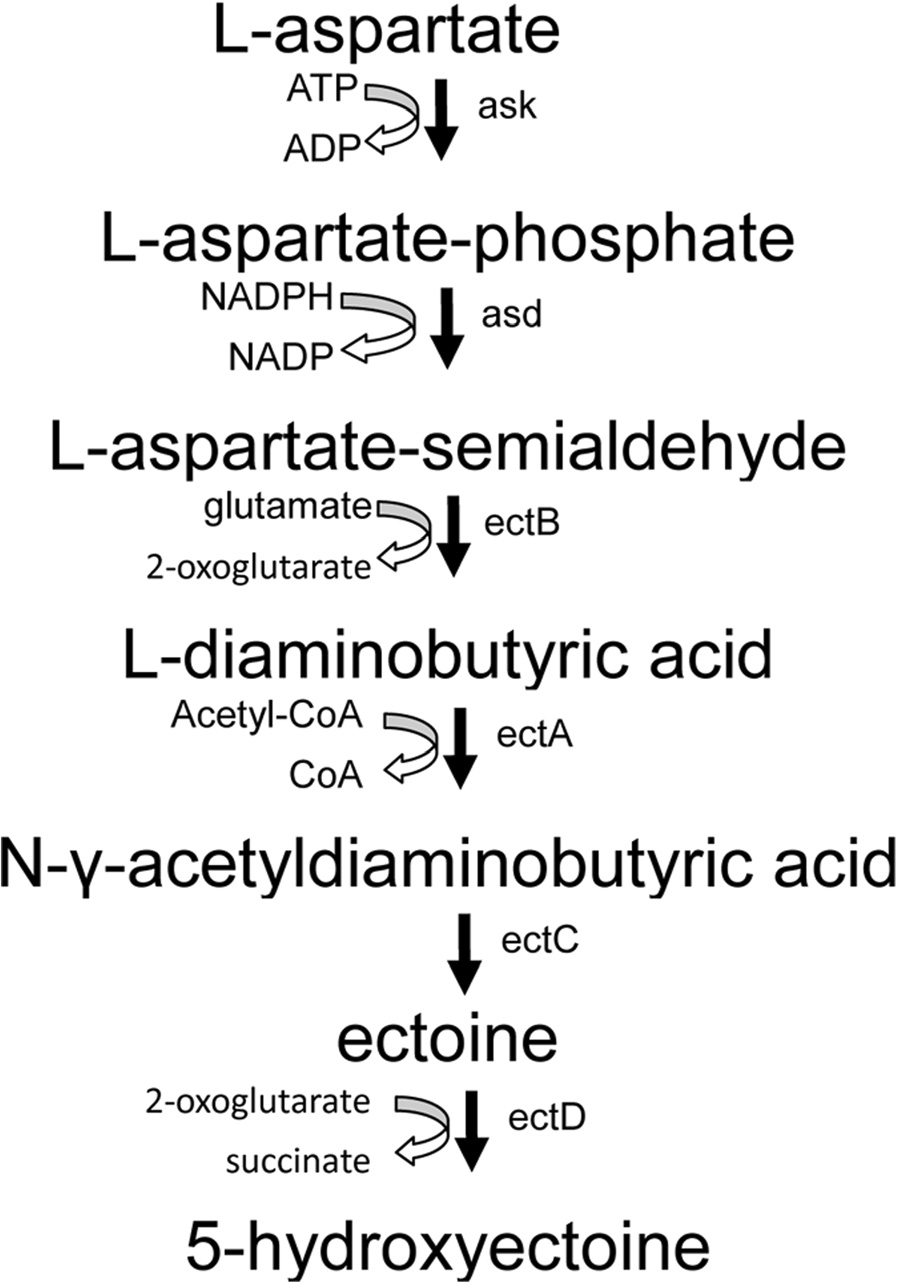
Biosynthetic pathway of ectoine in *H. elongata.* Ask: aspartate kinase; Asd: L-aspartate-beta-semialdehyde-dehydrogenase; EctB: L-2, 4-diaminobutyric acid transaminase; EctA: DABA-N-γ-acetyltransferase; EctC: ectoine synthase; EctD: ectoine hydroxylas.

To survive in salty environments, halophilic organisms have developed two different strategies to maintain osmotic balance. Some halophiles accumulate inorganic salts in the cytosol to counterbalance the high extracellular salt concentrations [1,2]; while some halophiles synthesize small organic molecules called compatible solutes as osmotic counterweights [3]. So far, many types of compatible solutes have been found, which can be divided into different classes by their structures, such as sugars, polyols, methylamines, betaines, amino acids and their derivatives. Ectoine (1, 4, 5, 6-tetrahydro-2-methyl-4-pyrimidinecarboxylic acid), a heterocyclic amino acid, is one of the most widely used compatible solutes in nature. It was discovered originally in the extremely halophilic phototrophic bacterium *Ectothiorhodospira halochloris* [4]. In addition to be functional as an osmotic counterweight, ectoine was found to be capable of protecting proteins, nucleic acids, cell membrane and even the whole cells against denaturation caused by heating, freezing, drying, or chemical agents [5-7]. For these properties, ectoine is currently produced for versatile commercial applications in the pharmaceutical industry. It can also be used as protein stabiliser, PCR enhancer, drying protective agent for microorganisms, and cosmetic additive [8-10]. The biosynthetic pathway of ectoine has been fully elucidated [11,12]. It shares the first two enzymatic steps with the biosynthesis of amino acids of the aspartate family: the synthesis of L-aspartate-phosphate through the ATP-dependent phosphorylation of L-aspartate by aspartate kinase (Ask), and the synthesis of L-aspartate-beta-semialdehyde through an NADPH-dependent reaction by L-aspartate-beta-semialdehyde-dehydrogenase (Asd). Ecotine is then formed by a specific route composed of three reactions. L-aspartate-beta-semialdehyde is first transaminated to generate L-2, 4-diaminobutyric acid (DABA) by DABA transaminase (EctB), which is then converted to N-γ-acetyldiaminobutyric acid by DABA-N-γ-acetyltransferase (EctA). A cyclic condensation reaction catalysed by ectoine synthase (EctC) finally leads to the formation of tetrahydropyrimidine L-ectoine. In some organisms, ectoine can be further converted to hydroxyectoine by ectoine hydroxylase (EctD) (Figure 1). So far, the *ectABC* gene cluster involved in the biosynthesis of ectoine has been founded and characterised from many microorganisms [13].

It was reported that many halophilic and halotolerant microorganisms could synthesize ectoine in salt stress environments [10]. To meet the increasing commercial demand of ectoine, the bacterial processes for ectoine production has been improved in the past two decades. A technical bioprocess called “bacterial milking” was developed using the halophilic eubacterium *H. elongata* [14], which involved a cyclic increase and decrease of the salt concentration for ectoine synthesis and secretion, respectively. By this process the maximum yield of ectoine could reach 155 mg/g DCW. In another work, Fallet et al. integrated the process of synthesis and secretion by utilising two continuous bioreactors. Through process optimisation, the yield of ectoine and hydroxyectoine reached 540 and 400 mg/g DCW respectively, which should be the highest level reported so far [15]. Although “bacterial milking” is currently the most successful method for ectoine production, some drawbacks of this method is inevitable including the corrosion of equipment, the reduction of cell growth rate and the difficulty of downstream processing due to the discontinuous production scheme and high salt concentration [16]. To overcome these shortages, *Halomonas salina*, a halophilic bacterium that can grow and excrete ectoine in a lower salt concentration, was chosen for ectoine production using monosodium glutamate as a carbon and nitrogen source in 0.5 M NaCl batch fermentors. An ectoine titre of 6.9 g/L was obtained, and the productivity was 7.9 g/L/day [17]. In addition, heterologous expression of the ectoine biosynthetic pathway in *E. coli* has been developed as an alternative strategy. Multiple ectoine biosynthetic gene clusters from different halophiles were introduced into *E. coli* [18-21]. When those ectoine clusters were under the control of the natural regulatory promoters, production of ectoine could only be detected in the recombinant *E. coli* strains in cells, but not extracellularly. The ectoine genes of *Chromohalobacter. salexigens* were expressed in *E. coli* DH5α under the control of an inducible *tet* promoter. After an induction time of 160 h in bioreactor cultivation, the recombinant *E. coli* strain could synthesis 6 g/L ectoine with a space-time yield of 40 mg/L/h, in which the vast majority of ectoine was excreted [16]. Recently, ectoine synthesis clusters from halophiles were also expressed in other model microorganisms. A microbial cell factory was obtained by introduction of a codon-optimised *ectABCD* gene cluster from *Pseudomonas stutzeri* into *Corynebacterium glutamicum*, which could produce ecotine at 6.7 g/L/day [22]. In another report, four hydroxyectoine biosynthesis genes from *H. elongata* were integrated into the genome of *Hansenula polymorpha*, and hydroxyectoine synthesis at the gram per litre scale was achieved [23].

In this study, a recombinant *E. coli* strain was constructed by overexpression of the *ectABC* gene cluster from *H. elongata* under the control of an inducible promoter. Using a whole-cell biocatalytic method, the recombinant cells could synthesize and secrete ectoine with high efficiency. The results highlight the promise of large-scale ectoine production with a more convenient and effective method.

## Results

### Cloning and expression of ectoine genes in *E. coli*

**Figure 2:**
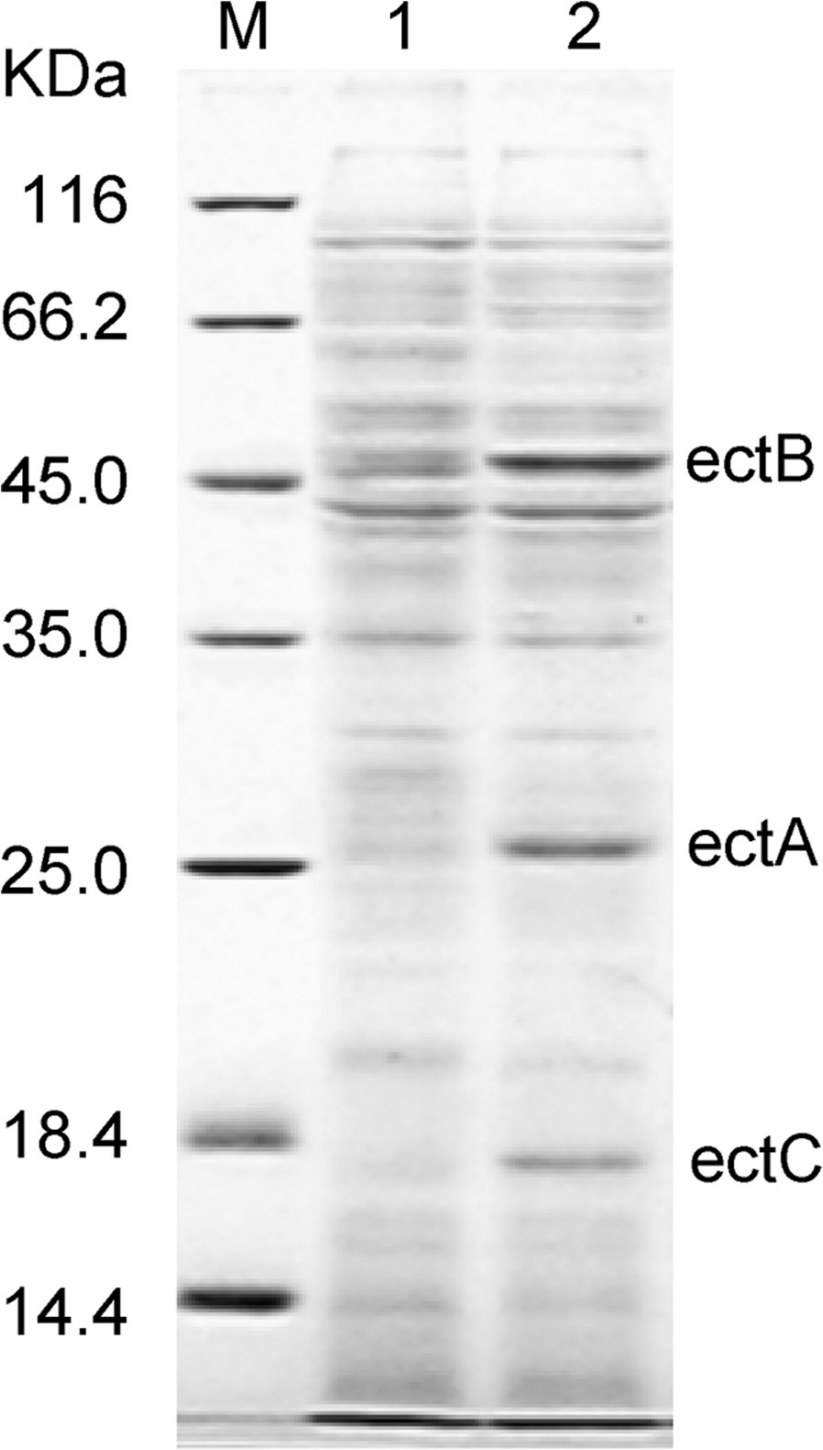
SDS-PAGE analysis of ectABC expressed in *E. coli*. M: protein molecular mass markers; 1: supernatant of cell extracts from induced BW (pBAD/HisA); 2: supernatant of cell extracts from induced BW (pBAD-ectABC).

The ectoine operon of *H. elongata* was cloned and sequenced. The sequence of the *ectABC* gene cassette was 2432 bp in length and consistent with the reported sequences. *E. coli* transformants harbouring pBAD-ectABC were induced with L-arabinose. SDS-PAGE of the cell lysate revealed three clear bands with molecular masses of 25 kDa, 48 kDa and 18 kDa, that were correspond to EctA, EctB and EctC from *H. elongata* respectively (Figure 2). Those bands were identified as EctA (77% sequence coverages, gi|503097538), EctB (67% sequence coverages gi|503097539) and EctC (79% sequence coverages gi|503097540) from *H. elongata* DSM 2581 by MALDI-TOF mass spectrometry, respectively. The results of SDS-PAGE and mass spectrometric indicated that the *ectABC* gene cassette from *H. elongata* was successfully expressed in *E. coli* K strain BW25113.

### Bioconversion and excretion of ectoine from recombinant *E. coli*

**Figure 3:**
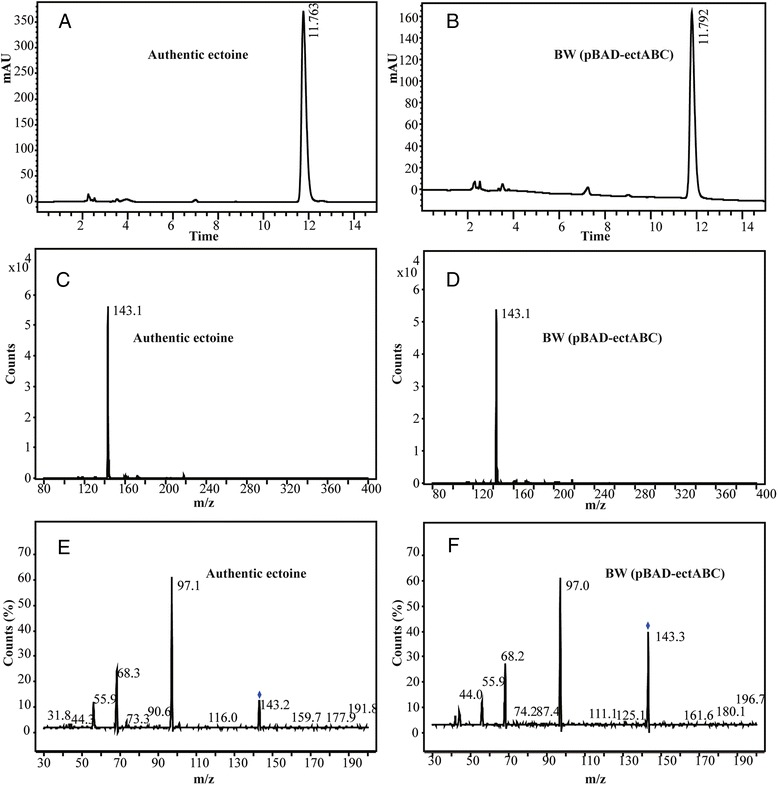
LC-MS analysis of ectoine from whole-cell biocatalysis. Panels **A** and **B**, Chromatograms obtained from authentic ectoine and from conversion products of BW (pBAD-ectABC) in HPLC analysis; Panels **C** and **D**, Spectra of two samples by LC-MS analysis; Panels **E** and **F**, Spectra of two samples by LC-MS/MS analysis.

Ectoine was successfully synthesized using a whole-cell biocatalytic method. In the reaction mixture, aspartate provided the substrate to synthesize L-aspartate-beta-semialdehyde, and glycerol could provide energy and the acetyl group for ectoine synthesis. KCl was added to the mixture to improve the activity and stability of ectB. Analyzed by HPLC, there appeared a new peak possessing the same retention time as the authentic ectoine in the tested samples (Figure 3A, B). After 24 h, the concentration of extracellular ectoine reached 1.56 mg/mL. To confirm that ectoine was really synthesized and excreted into the conversion solution, LC-MS and LC-MS/MS analyses were performed. Analyzed by LC/triple quadrupole mass spectrometry, for the compound in the conversion mixture, the retention time, the spectra signal of LC-MS anslysis (pseudo-molecular ion at *m/z* 143) (Figure 3C, D) and of LC-MS/MS analysis (product ions at *m/z* 143.3, 97.0, 68.2, 55.9, and 44.0) (Figure 3E, F) were all in agreement with the data of the authentic ectoine. Therefore, the compound in the conversion mixture was identified as ectoine.

### Optimization of ectoine bioconversion

**Figure 4:**
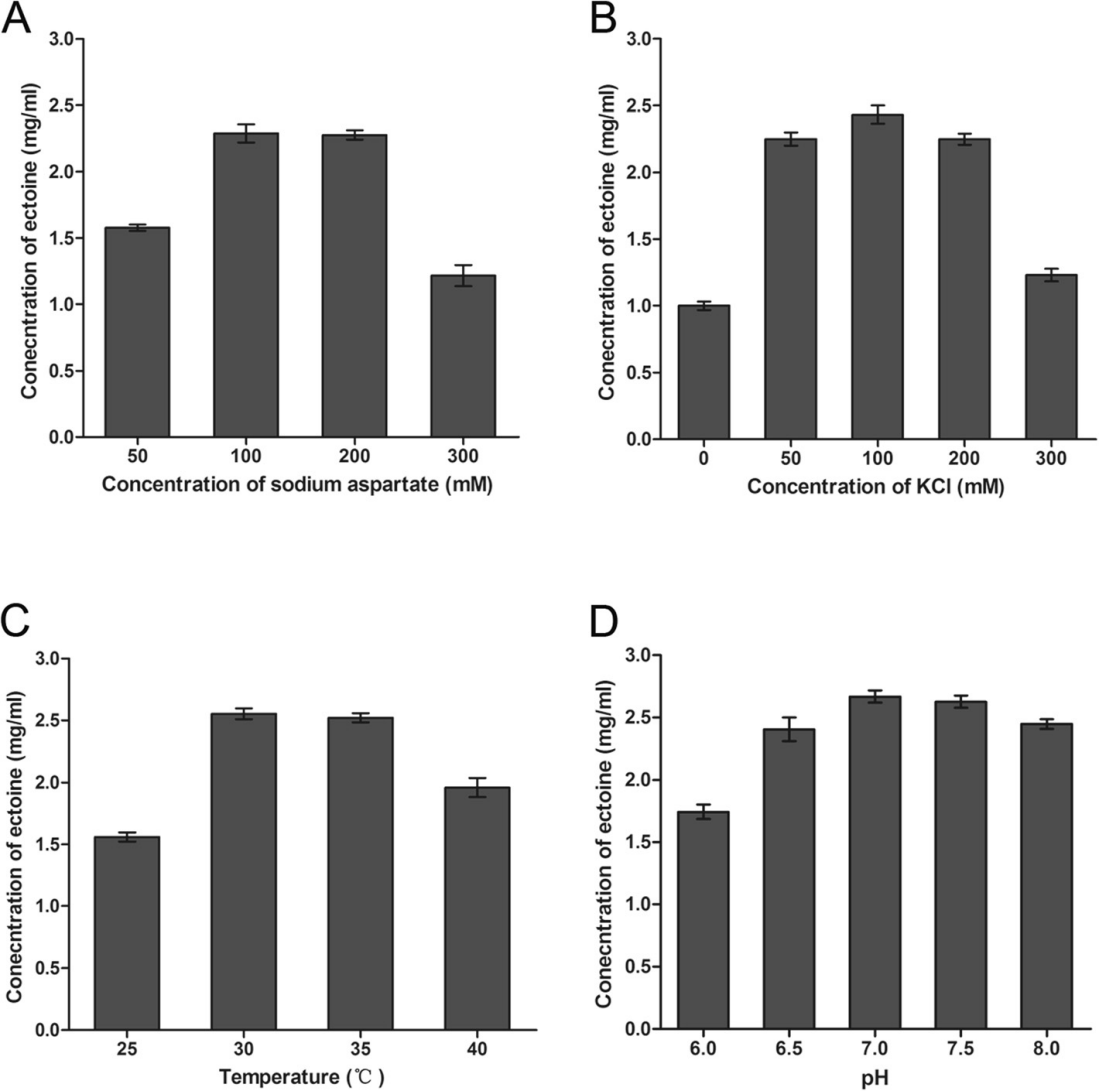
Optimization of ectoine production. The initial reaction mixture containing 100 mM sodium phosphate buffer (pH 7.0), 50 mM sodium aspartate, 50 mM KCl, 100 mM glycerol, and recombinant *E. coli* cells of 5 OD/mL was incubated at 30°C for 24 h. (**A**) Effects of sodium aspartate concentration on the production of ectoine. (**B**) Effects of KCl concentration on the production of ectoine (the concentration of sodium aspartate was 100 mM). (**C**) Effects of temperature on the production of ectoine (the concentrations of sodium aspartate and KCl were 100 mM). (**D**) Effects of pH on the production of ectoine (the concentrations of sodium aspartate and KCl were 100 mM). Error bars respresent standard deviations from triplicate biological replicates.

To determine the optimal conditions for ectoine production, multiple factors were investigated in whole-cell biocatalytic experiment in flask with a cell density of 5 OD/mL. The results of sodium aspartate concentration experiment indicated that the optimal concentration range of sodium aspartate was between 100 to 200 mM, over 2.25 mg/mL of extracellular ectoine was detected at these concentrations. Under a higher concentration of 300 mM sodium aspartate, only 1.23 mg/mL of ectoine was excreted into the mixture (Figure 4A). In KCl concentration comparative test, low concentration of KCl between 50 to 200 mM can significantly improve the production of ectoine. A maximum ectoine production (2.59 mg/mL) was obtained with a KCl concentration of 100 mM. However, in the presence of 300 mM KCl, the production of ectoine decreased sharply to only 1.25 mg/mL (Figure 4B). The effect of temperature was investigated between 25 to 40°C (Figure 4C). The results indicated that the highest yield was obtained at 30°C. The effect of pH on ectoine production processed at range of 6.0 ~ 8.0 indicated that the optimum pH was 7.0 (Figure 4D). In conclusion, the optimal reaction mixture in flask reaction was determined as follows: 100 mM sodium phosphate buffer (pH 7.0), 100 mM sodium aspartate, 100 mM KCl, and 100 mM glycerol to form a cell suspension (OD_600 nm_ = 5). The reactions were performed at 30°C, 200 rpm for 24 h. After the process optimization, the concentration of extracellular ectoine increased from 1.56 mg/mL to 2.67 mg/mL.

**Figure 5:**
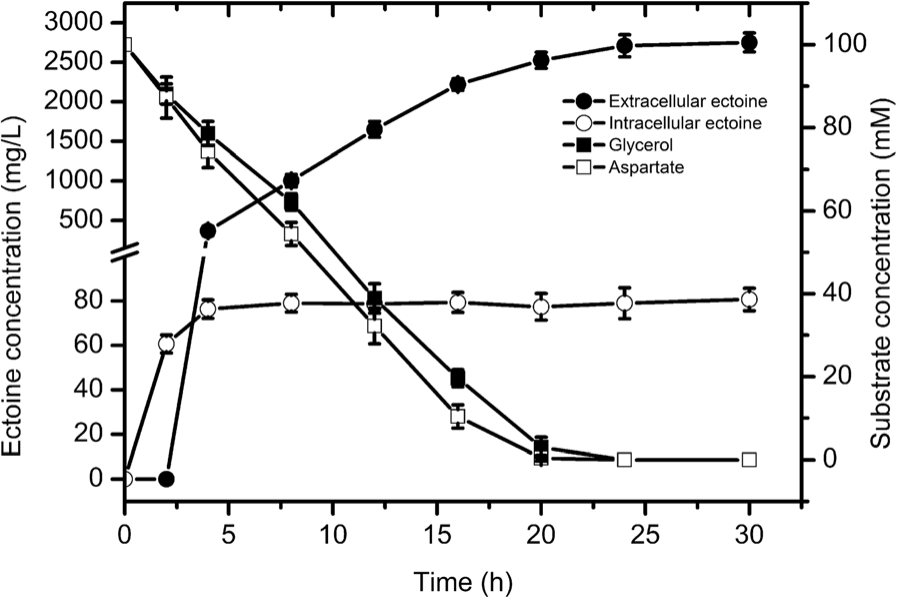
Time profiles of substrate concentration and ectoine production during flask-level bioconversion. An optimized reaction mixture containing 100 mM sodium aspartate, 100 mM glycerol, 100 mM KCl, 100 mM sodium phosphate buffer (pH7.0) and recombinant *E. coli* cells of 5 OD/mL was used. The bioconversion reactions were performed at 30°C and 200 rpm for 30 h. Error bars represent standard deviations from triplicate biological replicates.

In a flask reaction experiment using optimized conditions, the synthesis of ectoine and consumption of substrates during the whole bioconversion process were analysed in detail (Figure 5). The cells started to accumulate intracellular ectoine from the beginning of bioconversion, and after a reaction time of 4 h, the ectoine was beginning to be excreted; after 20 h, aspartate and glycerol were undetectable in the reaction mixture. After reacting for 24 h, the concentration of extracellular ectoine increased to 2.71 mg/mL. The corresponding extracellular ectoine concentration per gram dry cell weight reached 1748 mg/g DCW. During the whole reaction phase, the intracellular ectoine concentration was maintained at approximately 50 mg/g DCW.

### High production of ectoine using high density cells in a fermentor

**Figure 6:**
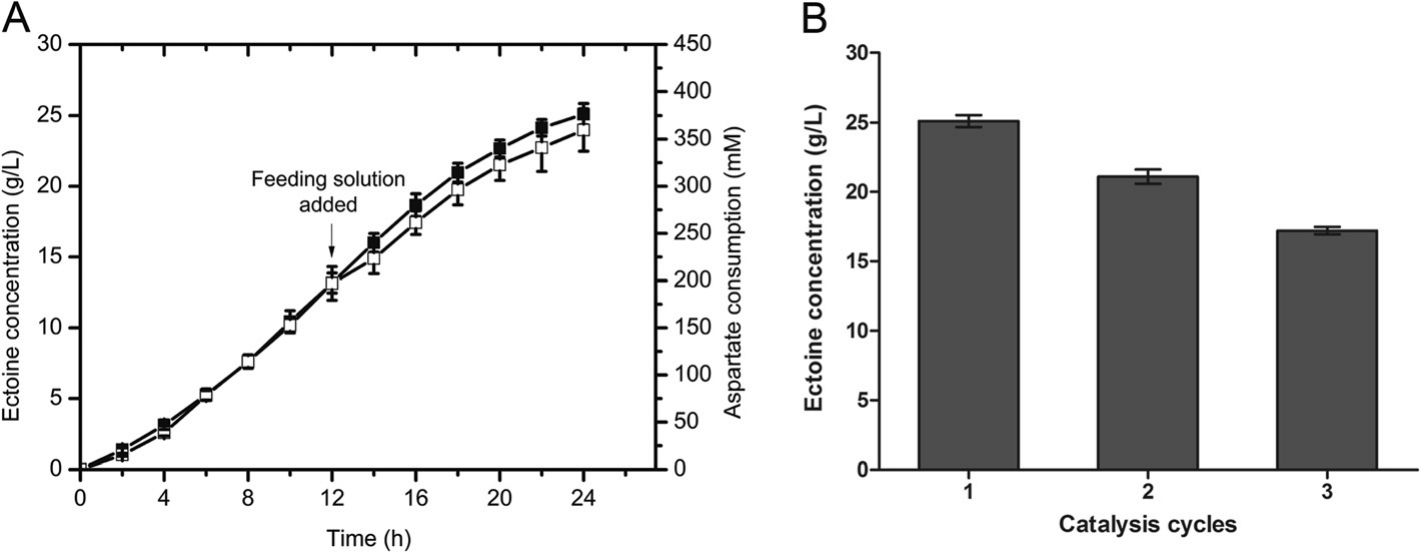
Ectoine production in fermentor. An initial reaction mixture containing 200 mM sodium aspartate, 200 mM glycerol, 100 mM KCl, 100 mM sodium phosphate buffer (pH7.0), and recombinant *E. coli* cells of 20 OD/mL was used. (**A**) Time profiles of aspartate consumption and ectoine excretion in fermentor. Legend: excreted ectoine (*black squares*), aspartate (*white squares*). After 12 h of bioconversion the feeding solution was added (↓) with a flow speed of 25 mL/h. (**B**) Repeated use of cells to synthesize ectoine in fermentor. Error bars respresent standard deviations from triplicate biological replicates.

Ectoine bioconversion was also performed with higher density cells (OD_600 nm_ = 20) in a fermentor (Figure 6A). When the reaction proceeded for 12 h, the aspartate and glycerol were all consumed and feeding solution was added into the reaction system with a flow speed of 25 mL/h. During the reaction phase, a significant quantity of ectoine was synthesized and excreted at a rate of 1046 mg/L/h. After a reaction time of 24 h, the ectoine concentration reached 25.1 g/L in the medium with a productive yield of 4048 mg/g DCW, which was significantly higher than that in flask reactions with low density cells (OD_600 nm_ = 5). The ectoine bioconversion was successfully amplified in a fermentor, the yield of ectoine increased at least nine times compared with that in flasks.

### Production of ectione by multiple rounds of whole-cell biocatalysis

To test whether the cells in the whole-cell biocatalysis could be used by multiple rounds, the process of whole-cell biocatalysis using aspartate and glycerol as substrates was repeated for another two cycles with the same batch of *E. coli* cells. The results demonstrated that the extracellular ectoine concentrations of second and third round were 21.1 g/L and 17.2 g/L, respectively. In other words, 84% and 69% of yields were achieved for the second and the third round of catalysis as compared to the first round, respectively (Figure 6B). Thus, multiple rounds of whole-cell biocatalysis are cost effective to further improve the production of ectione.

## Discussion

In recent decades, whole-cell biocatalysis has been widely applied as an alternative to chemical methods for large-scale synthesis of chemicals because it is more environmentally friendly. Whole-cell biocatalysis allows cascades of enzymatic reactions that involve multiple enzymes, cofactors, and substrates; it can also help stabilise enzymes with the protective nature of cell envelopes and makes cofactor regeneration much easier [24]. *E. coli* K12 is often used as a suitable cell factory in bioconversion because it is a nonpathogenic strain with a well-studied genetic background and a powerful genetic tool system for metabolic engineering [25]. With a relatively well-developed fermentation, *E. coli* K12 is also easy to grow because of its short generation time and ability to synthesize everything needed for generating a completely new cell from low raw materials. Most advantageously, the *E. coli* genome sequence lacks an ectoine catabolic pathway [26], eliminating product degradation due to the reuse of ectoine as a carbon or nitrogen source. The biosynthesis of ectoine from L-aspartate is a complex process containing multiple enzymatic reactions in microorganisms. Using the above whole-cell biocatalytic method for the production of ectoine by recombinant *E. coli* has the potential to become the most promising method for commercial production.

As an industrial strain for ectoine production, the ectoine synthesis gene cluster of *H. elongata* has been cloned for more than 10 years [27], and the characteristics of ectoine biosynthetic enzymes have been described in detail [28]. However, there were still no reports describing heterologous ectoine synthesis in *E. coli* using the *ectABC* genes from this industrial strain. In our experiment, the *ectABC* gene cluster was first introduced into *E. coli* K strain BW25113 using the expression plasmid pBAD/HisA. Under the control of the *ara* promoter, all three enzymes corresponding to ectoine synthesis achieved soluble expression. Whole-cell biocatalysis was introduced into ectoine synthesis using aspartate and glycerol as substrates. The bioconversion was performed in shaking flasks and in a fermentor with cell concentrations of 5 OD/mL and 20 OD/mL, respectively. In the fermentor the extracellular ectoine concentration reached 25.1 g/L with a productive yield of 4048 mg/g DCW, which is markedly higher than the yield in the flask reaction of 1748 mg/g DCW. By controlling the pH and substrate feeding, the cell catalysis efficiency can be significantly improved in fermentor. The highest yield in history was achieved in *C. salexigens* (540 and 400 mg/g DCW for ectoine and hydroxyectoine respectively) [15], which is significantly lower than our ectoine yield (4048 mg/g DCW). After the first round of bioconversion, the cells were reused two more times, and a total yield of 63.4 g ectoine was obtained using one litre cells.

Ectoine is synthesized from aspartate-semialdehyde, the central intermediate in the synthesis of amino acids belonging to the aspartate family. Therefore, in recombinant *E. coli*, the synthesis rate of aspartate-semialdehyde determines the efficiency of ectoine conversion [18]. In the ectoine bioconversion system, sodium aspartate was added to the reaction mixture, and under the catalysis of *E. coli* endogenous Ask and Asd, aspartate could be converted to aspartate-semialdehyde more effectively than other substrates. In addition, under catalysis by glutamate-aspartate aminotransferase the amino group of aspartate could also be transferred to glutamate, which could provide the amino group for the transamination catalysed by EctB, the first reaction of the specific route for ectoine synthesis. The 2-oxoglutarate converted from glutamate by EctB could further supply substrate for the glutamate-aspartate transamination reaction to form glutamate. In summary, aspartate should be an ideal substrate for ectoine bioconversion because it can be used as both a synthetic precursor and an amino donor.

Another factor that may affect the efficiency of ectoine synthesis is the protein expression level of ectoine synthesis enzymes. In our experiment, under the control of the *ara* promoter all three enzymes of EctABC achieved overexpression, but in other reports with lower ectoine synthesis, not all of these three enzymes were detectable by SDS-PAGE [16,20]. We have also used pET28a with the T7 promoter to express EctABC in Bl21 (DE3), and in SDS-PAGE analysis only EctA and EctC were detectable, and there were no clear bands corresponding to EctB. The ectoine yield from the pET expression strain was only about one-third that of the pBAD expression strain (data not shown in this paper).

Our research confirmed that transgenic *E. coli* could efficiently synthesize and excrete ectoine into the medium. Although multiple ectoine biosynthetic genes have been expressed in *E. coli*, a similar finding has only been reported by Schubert et al. In their experiment, a high density culture of 20 g DCW/L was obtained in a fermentor. Following induction with anhydrotetracycline, they observed continuous excretion of ectoine at a rate of 2 mg/g DCW/h, while the cellular level of ectoine stayed low at 5 mg/g DCW [16]. In our research, a lower density of cells with 6.45 g DCW/L (OD_600 nm_ = 20) was used to produce ectoine in a fermentor. During the synthetic phase, the average excretion rate of ectoine was 26.1 mg/g DCW/h, whereas the cellular ectoine stayed at 50 mg/g DCW. Although *E. coli* does not synthesize ectoine, it was found that *E. coli* can pump ectoine out efficiently. At first, it was speculated that the proline transport systems, ProP and ProU, responsible for ectoine uptaking may also be functional in its excretion. However, the ProP and ProU deficient mutants could also pump ectoine out, indicating that there should be other efflux systems participating this process [26]. Moreover, Schubert et al. excluded the possibility that *E. coli* excretes ectoine by the unspecific mechanosensitive channels [16]. Further studies are demanded to elucidate the excretion mechanism of ectoine in *E. coli*.

## Conclusion

The gene cluster of ectoine biosynthesis from the halophilic bacterium *H. elongata* was introduced into *E. coli* K strain and a powerful whole-cell biocatalytic system was constructed. Using aspartate and glycerol as the direct substrates, a large amount of ectoine was synthesized and excreted into the medium during the course of whole-cell biocatalysis. After the process optimization, the ectoine production increased from 1.56 to 2.67 mg/mL in flask. In fermentor, the ectoine production reached 25.1 g/L in 24 h, with a productive yield of 4048 mg/g DCW, which is the highest value ever reported. The cells can be used for at least 3 rounds and a total yield of 63.4 g ectoine was obtained using one litre cells. Our study herein provided a feasible biosynthesis process of ectoine with potential applications in large-scale industrial production.

## Methods

### Bacterial strains and media

*H. elongata* DSM 2581 (CGMCC No.1.6329) was grown aerobically in Luria-Bertani (LB) medium consisting of 1.0% tryptone, 0.5% yeast extract, and 1% NaCl, modified by the addition of NaCl at a final concentration of 15% (wt/vol). *E. coli* DH5α was used as a host in general gene cloning and the construction of recombinant plasmids. *E. coli* K12/BW25113 (*rrnB3* Δ*lacZ4787 hsdR514* Δ*(araBAD)567* Δ*(rhaBAD)568 rph-1*) was used as the host strain for protein expression and ectoine production. LB medium was used for growing *E. coli* cells. A defined medium (DM) was used for fed-batch cultures. This medium contains (per litre) 10 g glucose, 8 g of (NH_4_)_2_HPO_4_, 13.3 g of KH_2_PO_4_, 1.2 g of MgSO_4_ · 7H_2_O, 1.7 g of citric acid, and 10 mL of a trace metal solution that contains (per litre of 5 M HCl) 10 g of FeSO_4_ · 7H_2_O, 2.25 g of ZnSO_4_ · 7H_2_O, 1 g of CuSO_4_ · 5H_2_O, 0.5 g of MnSO_4_ · 5H_2_O, 0.23 g of Na_2_B_4_O_7_ · 10H_2_O, 2 g of CaCl_2_ · 2H_2_O, and 0.1 g of (NH_4_)_6_MO_7_O_24_. The antibiotic ampicillin (100 mg/L) was used to maintain the heterologous plasmid in the genetically modified strain.

### Molecular biological methods

Standard methods were used for genomic DNA and plasmid DNA extraction, PCR, ligation, plasmid construction, and transformation [29]. The Pfu DNA polymerase, restriction endonucleases, T4 DNA ligase and vector pMD19-T Simple were purchased from Takara Bio (Takara Biotechnology dalian Co. Ltd., China). The vector pMD19-T Simple was used for cloning of PCR products and pBAD/HisA (Invitrogen, USA) was used for gene expression.

The *ectABC* gene cluster was amplified by PCR using genomic DNA extracted from *H. elongata* DSM 2581 as the template and primers P1 and P2 as follows: P1, 5′-CCTA*GCTAGC*ATGAACGCAACCACAGAGCCCTTTA-3′; P2, 5′-CCG*GAATTC*TTACAGCGGCTTCTGGTCGTCGGCT-3′ (*NheI* and *EcoRI* sites are in italics). The full length ectoine operon containing the genes *ectA*, *ectB* and *ectC* was amplified without any terminator, operator, or promoter region. The PCR products were ligated into the cloning vector pMD19-T Simple and verified by sequencing. The recombinant vector was transformed into *E. coli* DH5α. The *NheI*-*EcoRI* fragment from the recombinant cloning vector containing the *ectABC* gene cluster was recloned into the expression plasmid pBAD/HisA under the control of the *ara* promoter. The resulting vector pBAD-ectABC was transformed into *E. coli* BW25113.

### Protein expression and identification

The recombinant strain BW (pBAD-ectABC) was grown at 37°C in LB broth with ampicillin. When the optical density of the culture at 600 nm reached 0.6, L-arabinose was added at a final concentration of 0.1% and incubated at 30°C for 6 h. The cells were harvested by centrifugation and resuspended in 50 mM of potassium phosphate buffer (pH 7.0). The cell suspension was sonicated (Soniprep 150 sonifier) and centrifuged (12,000 × g, 10 min). The supernatant was subjected to SDS-PAGE analysis. For mass spectrometric protein identification, bands of interest were excised and submitted to in-gel reduction, alkylation, and digestion with bovine trypsin (12.5 ng/μl, sequencing grade; Roche Applied Science) as previously described by Sechi and Chait [30]. Afterward, the trypsinized peptides were analysed on an Applied Biosystems 4700 Proteomic Analyzer (Applied Biosystems, Framingham, MA) as previously described [31]. The resulting MS and MS/MS data were analyzed and peak lists were generated using GPS Explorer 3.5 (Applied Biosystems) and were further searched using MASCOT 2.0 search engine (MatrixScience, London, U.K.) against SwissProt protein sequence database download in Dec, 2014 from NCBI.

### Bioreactor cultivation

Precultures of recombinant strain BW (pBAD-ectABC) were prepared in shaking flasks (30°C, 200 rpm) with LB medium containing ampicillin. Fed-batch cultivations were performed in a 6.6 litre jar fermentor (Bioflo 3000; New Brunswick Scientific Co., Edison, N.J.) containing 2.7 litres of DM at 30°C. The pH was controlled at 7.0 ± 0.05 by automatic feeding of 25% (v/v) NH_4_OH. The dissolved oxygen concentration was maintained above 20% air saturation by supplying air at 1 vvm (air volume/working volume/minute) and by automatically controlling the agitation speed up to 1000 rpm. The feeding solution contained 500 g of glucose and 10 g of MgSO_4_ · 7H_2_O per litre was added periodically after glucose depletion. Expression of ectoine genes were induced for 8 hours by adding L-arabinose to the final concentration of 1 g/L when the OD_600_ value had reached 30.

### Bioconversion conditions

After induction, cells were collected from shaking flask cultures by centrifugation at 6,000 × g for 20 min, washed with 0.85% NaCl solution twice, and then resuspended in the reaction mixture containing 100 mM sodium phosphate buffer (pH 7.0), 50 mM sodium aspartate, 50 mM KCl, and 100 mM glycerol to form a cell suspension (OD_600 nm_ = 5). The bioconversion reactions were performed using cell suspensions in 100-mL flasks in which the final liquid volume was 30 mL at 30°C and 200 rpm on a rotary shaker for 24 h.

To optimize the component of reaction mixture, various concentrations of sodium aspartate (50, 100, 200 and 300 mM) were compared in bioconversion test. K^+^ has been reported that can improve the activity and stability of ectB [28], so various concentrations of KCl (0, 50, 100, 200 and 300 mM) were added into the reaction mixture to investigate the effect on ectoine production. To evaluate the effect of temperature on ectoine bioconversion, the reactions were performed at 25, 30, 35 and 40°C at pH 7.0. To estimate the effect of pH, the reaction mixture were adjusted to pH levels of 6.0, 6.5, 7.0, 7.5, and 8.0, and incubated at 30°C.

The bioconversion of ectoine was also carried out in a fermentor. Cells after induction from a fed-batch fermentation were harvested by centrifugation at 6,000 × g for 20 min, washed with 0.85% NaCl solution twice, and then resuspended in the reaction mixture containing 100 mM sodium phosphate buffer (pH 7.0), 200 mM sodium aspartate, 200 mM glycerol and 100 mM KCl to form a cell suspension (OD_600 nm_ = 20). Three litres of cell suspension was added into a 6.6 litre fermentor. NH_4_OH (2 M) and HCl (2 M) were used to keep the pH at 7.0 ± 0.05. The reaction was performed at 30°C with agitation speed of 600 rpm and the aeration rate at 1 vvm. The residual aspartate and glycerol were monitored by HPLC. When the substrates were consumed, feed solution containing 2 M sodium aspartate, 2 M glycerol and 100 mM KCl was added periodically according to the rate of substrate consumption. Subsequently, the cells were re-harvested by centrifugation and a second round of synthesis was performed in the same reaction mixture. This process was repeated for two rounds.

### Cell biomass measurement

Biomass concentrations were estimated by measuring the optical density at 600 nm (OD_600_). The dry cell weight (DCW) was calculated on the basis of OD_600_ (1 OD/mL = 0.31 g DCW/L). Glucose concentrations were measured by a SBA-40E biosensor analyser (Institute of Biology, Shandong Province Academy of Sciences, China).

### HPLC analytical methods

For the identification of intracellular ectoine, cells were harvested by centrifugation and freeze-dried. Approximately 20 mg of cell material was extracted according to the method of Bligh and Dyer [32]. The extracellular concentration of ectoine was directly measured after dilution of the samples with acetonitrile/water (70:30 v/v). The intracellular and extracellular samples were filtered through a 0.22-um pore size membrane filter and then analysed by isocratic HPLC (Agilent 1260 series, Hewlett-Packard) using an Agilent ZOBAX NH2 column (2.6 × 250 mm, 5 um) with acetonitrile/water (70:30 v/v) at a flow rate of 1 mL/min as the mobile phase. UV detection at 210 nm was used to measure ectoine, and simultaneously a refraction index detection system was used to measure glycerol at 35°C. The aspartate concentration was determined by reversed phase HPLC according to an Agilent method [33]. The samples were automatically derivatized with OPA and then injected into a Zorbax Eclipse-AAA column (4.6 × 75 mm, 3.5 μm) at 40°C with detection at 338 nm. The retention times of ectoine, glycerol and aspartate were determined by using commercially available samples (Sigma).

### LC-MS analysis of ectoine

The ectoine purified by HPLC on an Agilent ZOBAX NH_2_ column described above was indentified using an Agilent 1260/6460 high performance liquid chromatography/triple quadrupole mass spectrometer (Agilent, USA) using ESI source in positive ionization mode. The chromatographic separation was carried out using a ZORBAX SB-Aq (100 mm × 2.1 mm, 3.5 μm, Agilent, USA) at 30°C. The mobile phases were the mixture of 95% methanol and 5% water (containing 0.1% formic acid) (v/v) under isocratic elution program at a flow rate of 0.2 mL/min. For the LC-MS analysis, the mass scan ranged from *m/z* 80 to 400. For the LC-MS/MS analysis, the product ion scan of *m/z* 143 was performed with collision energy of 17 V. The fragmentor voltage was 110 V. The capillary voltage and atomizing gas pressure were 4.0 kV and 35 psi, respectively. The flow rate of drying gas was 12 mL/min and the temperature of solvent removal was 350°C. Nitrogen was used as collision gas.
